# Ag-Modified In_2_O_3_/ZnO Nanobundles with High Formaldehyde Gas-Sensing Performance

**DOI:** 10.3390/s150820086

**Published:** 2015-08-14

**Authors:** Fang Fang, Lu Bai, Dongsheng Song, Hongping Yang, Xiaoming Sun, Hongyu Sun, Jing Zhu

**Affiliations:** 1 Beijing National Center for Electron Microscopy, School of Materials Science and Engineering, The State Key Laboratory of New Ceramics and Fine Processing, Key Laboratory of Advanced Materials (MOE), Tsinghua University, Beijing 100084, China; E-Mails: ff99@mail.tsinghua.edu.cn (F.F.); todongsheng@126.com (D.S.); hpyang1989@163.com (H.Y.); 2 National Center for Nanoscience & Technology University of Chinese Academy of Sciences, No. 11 First North Road, Zhongguancun, Beijing 100190, China; E-Mail: whitelu@mail.tsinghua.edu.cn; 3 State Key Laboratory of Chemical Resource Engineering, Beijing University of Chemical Technology, Beijing 100029, China; E-Mail: sunxm@mail.buct.edu.cn

**Keywords:** hierarchical porosity, In_2_O_3_/ZnO, conductivity, negative curvature, formaldehyde sensing

## Abstract

Ag-modified In_2_O_3_/ZnO bundles with micro/nano porous structures have been designed and synthesized with by hydrothermal method continuing with dehydration process. Each bundle consists of nanoparticles, where nanogaps of 10–30 nm are present between the nanoparticles, leading to a porous structure. This porous structure brings high surface area and fast gas diffusion, enhancing the gas sensitivity. Consequently, the HCHO gas-sensing performance of the Ag-modified In_2_O_3_/ZnO bundles have been tested, with the formaldehyde-detection limit of 100 ppb (parts per billion) and the response and recover times as short as 6 s and 3 s, respectively, at 300 °C and the detection limit of 100 ppb, response time of 12 s and recover times of 6 s at 100 °C. The HCHO sensing detect limitation matches the health standard limitation on the concentration of formaldehyde for indoor air. Moreover, the strategy to synthesize the nanobundles is just two-step heating and easy to scale up. Therefore, the Ag-modified In_2_O_3_/ZnO bundles are ready for industrialization and practical applications.

## 1. Introduction

Air pollution, especially indoor air pollution, with respect to public health has been realized by more and more people, and enhanced the requirement for highly sensitive toxic gas sensor monitors as well. Formaldehyde (HCHO), as one of the typical indoor air pollutions, is highly toxic to all animals and human beings even at concentrations as low as 0.1 ppm [1]. Therefore, effective methods to detect low concentration HCHO are of great importance and in huge demand in practical applications. Compared to the traditional detect method, semiconductor-based gas sensor, especially metal oxides, has been widely applied in public safety and environmental monitoring with their advantages of low cost, short response time, and good stability [2,3]. The fundamental mechanism of semiconductor gas sensors is based on surface-chemical interaction between detected gas molecules and the surface atoms of the sensor material, which changed the whole conductivity of sensor. In that case, by adjusting the surface component crystal structure or the surface morphology of sensor materials, different kinds of gas sensor can be obtained [4,5,6,7]. As for those facts, nanocomposite materials with tunable chemical components and tailored surface structures are effective for improving gas sensibility, selectivity and detected limitation. Firstly, desired electric structure with enhanced electric and photoelectric properties can be prepared by changing sensor materials` chemical composition. Then, nanomaterials, especially three dimensional (3D) micro/nano nanomaterials composed by small nanoparticles have high surface area and atom steps, which containing lots of dangling bonds and low-coordination atoms are active for surface reaction and thus catalysis [8,9,10,11,12,13,14,15,16,17,18].

Various nanocomposites such as In_2_O_3_/ZnO, SnO_2_/ZnO, In_2_O_3_/Ag_2_O, In_2_O_3_/SnO_2_, have been used for sensitive and selective gas sensors [19,20,21,22,23,24]. In our former work, we reported a porous In_2_O_3_-based HCHO sensor with the sensitivity low to 100 ppb, which was health standard limitation on the HCHO concentration in air [25]. However, as one of the typical scarce metals, the commercialization and popularization of indium-based device has been limited by the high price of raw materials. Comparably, ZnO with cheaper price, as an important semiconductor, has been developed by many scientists with diversity morphology and has been widely used in many fields. Hence, In_2_O_3_/ZnO nanocomposite was thought to be applicable for gas sensor detection [19]. However, the detected gas limitation of the reported sensors based on In_2_O_3_/ZnO were still still too high for industrial application. As for HCHO-sensing, low gas detection limits were thought to be the most important thing because of the toxicity [1]. Although significant progresses with the lowest HCHO-detection limits down to 500 ppb have been achieved in the literature [5], the limits are still higher than 100 ppb, which was the health standard for human beings [1]. At the same time, the sensors based on Ag-loaded In_2_O_3_ hierarchical nanostructures show fast response time (0.9 s), recovery time (14 s), high sensitivity and good sensing selectivity for 20 ppm HCHO, which showed quick response for HCHO [22]. In that case, a HCHO sensor with Ag-loaded In_2_O_3_/ZnO porous nanostructure is thought to be sensitive with quick response time and low gas detection limits.

In this paper, we designed and synthesized porous Ag-modified In_2_O_3_/ZnO bundles, which composed by many little In_2_O_3_/ZnO nanoparticles with diameter about 10 nm. The detect limit of the designed Ag-modified In_2_O_3_/ZnO bundles low to 100 ppb, which meet the health standard limitation on the HCHO concentration in air, and the response and recover times as short as 6 s and 3 s respectively. The surface atom structure and morphology were also analyzed using SEM, TEM and EDS.

## 2. Experimental Section

### 2.1. Synthesis of In_2_O_3_/ZnO Bundles

In a typical synthesis, In(NO_3_)_3_·6H_2_O of 2.546 g and Zn(NO_3_)_2_·6H_2_O of 0.991 g was quickly added and stirred in de-ionized (DI) water of 20 mL containing 9 g urea to form a clear solution. The solution was heated at 90 °C for 6 h and then cooled to room temperature, resulting in white precipitates in the solution. The white precipitates were separated from the solution by centrifugation at 2000 rpm (revolutions per minute) for 10 min, and were then washed with DI water and ethanol.

The as-prepared white precipitates were heated in air from room temperature to 100 °C by the rate of 1.3 °C /min, then to 400 °C by 2.2 °C /min, kept at 400 °C for 3 h and finally cooled to room temperature, and then, the light yellow In_2_O_3_/ZnO bundles was obtained, named Z 1.

### 2.2. Synthesis of Ag-modified In_2_O_3_/ZnO Bundles

In a typical synthesis procedure, AgNO_3_ of 0.085 g, In(NO_3_)_3_·6H_2_O of 1.909 g and Zn(NO_3_)_2_·6H_2_O of 0.744 g was quickly added and stirred in de-ionized (DI) water of 20 mL containing 7.550 g urea like the synthesis of Z 1. In addition, the solution was also heated at 90 °C for 6 h and then cooled to room temperature, resulting in light yellow precipitates in the solution. The precipitates were separated and washed with DI water and ethanol and then heated at 400 °C for 3 h. After the oven cooled to room temperature, the final product of Ag-modified In_2_O_3_/ZnO bundles was obtained, named Z 2.

### 2.3. Characterization

The morphologies, element analysis and structures of products were characterized by field emission scanning electron microscope (SEM, Hitachi-5500), conventional transmission electron microscope (TEM, Tecnai G^2^20 at 200 kV). The Specific surface areas (SSAs) were measured by the Bruauer-Emmett-Teller (BET) method with nitrogen adsorption-desorption isotherm.

### 2.4. HCHO-Sensing Measurement

As-prepared samples were mixed and ground with glycol in an agate mortar to form a paste. The paste was smeared evenly onto the alumina tube of a standard commercial sensor device purchased from Zhengzhou Winsen Electronics Technology, China (see more details of its schematic and optical images in Figure S1 of Supporting Information) [25]. After being coated with gas-sensing materials (such as the Ag-modified In_2_O_3_/ZnO bundles, Z 2), the sensor device becomes a ready gas sensor. The sensor devices were dried in air for a week, and the thickness of the sample coating was 1.5 µm. Then, the sensor were connected to the sensor holder of a WS-30A measuring system (Zhengzhou Winsen Electronics Technology, Zhengzhou, China) and the sensor was placed in the test chamber of the measuring system. The chamber can supply an isolated environment to the target gas, HCHO. In the beginning of the HCHO-sensing measurement, the sensor device was aged for 2 h by heating it at 100 °C and 300 °C [20,25], the aging process could not effect the surface morphology of our samples which were proved by SEM images shown in Figure S2. After the ageing, some HCHO solution was injected onto the quickly-evaporating heater, and the change in the reading of the voltage meter of the measuring system was monitored. The volume of the testing chamber was 18,000 mL, and the liquid formaldehyde solution was injected into the chamber by an injector from a small hole in one side of the chamber. After the formaldehyde solution was injected, the solution would be evaporated immediately by a built-in heater to become HCHO gas, the HCHO concentration was calculated with the volume of the liquid formaldehyde solution and the volume of the chamber.

## 3. Results and Discussion

**Figure 1:**
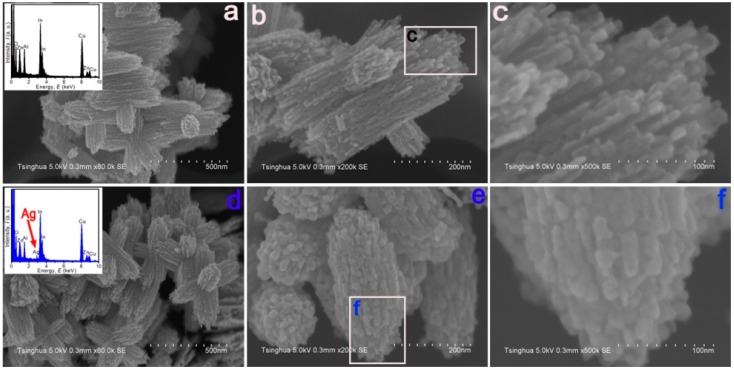
(**a**–**c**) Low- and high-magnification SEM images of Z 1, In_2_O_3_/ZnO bundles, the inset in (a) is the EDX pattern, and the image (**c**) is a close-up of the marked place in (**b**). (**d**–**f**) Low- and high-magnification SEM images of Z 2, Ag-modified In_2_O_3_/ZnO bundles, the inset in (**c**) is the EDX pattern, and the image (**f**) is a close-up of the marked place in (**e**).

**Figure 2:**
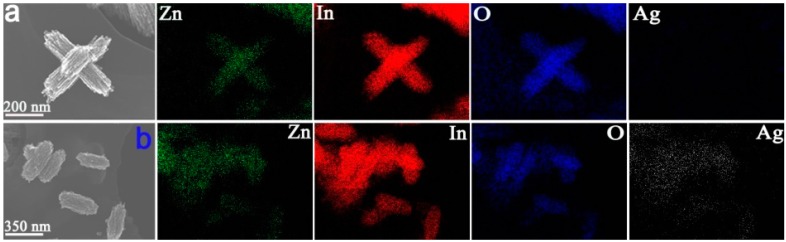
EDX mapping of the Zn, In, O and Ag elements in Z 1 and Z 2: (**a**) Z 1; (**b**) Z 2.

The surface morphologies of the as-prepared have been characterized by SEM as shown in Figure 1. With SEM images, it can be known that the morphology of Sample Z 1 was bundle-like composing by nanoparticles with the diameter of 10–30 nm, and the atom ratio of Zn and In was about 1:2 in agreement with that of the added mol ratio of Zn and In. Additionally, the morphology of sample Z 2 is similar to that of Z 1 shown in Figure 1 d, e and f, the surface structure of Z 2 is also consisting with nanoparticles with the diameter of 10–30 nm, and the atom ratio of Ag, Zn and In was about 1:5:10. From the images of these two samples, it can be indicated that the Ag modifying process didn’t affect the surface morphology of the final product. At the same time, from Energy-dispersive X-ray spectroscopy (EDX) analysis in Figure 1d, it can be known that Ag element was doped in In_2_O_3_/ZnO bundles in sample Z 2, which is further proved by EDX mapping of the Zn, In, O and Ag elements in Z 1 and Z 2 shown in Figure 2, in which Ag element is doped equally to sample Z 2. Nanogaps between each nanoparticle in these two samples let the surface be porous-structure, which may bring about higher surface area, better gas diffusion and more active sites [25].

**Figure 3:**
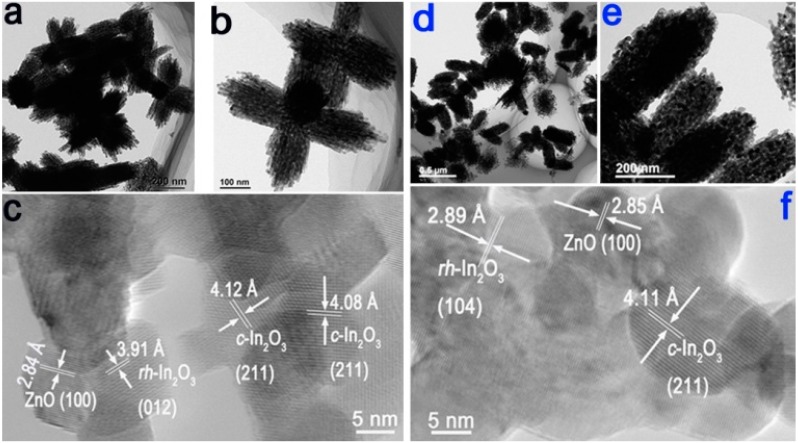
(**a**–**c**) Low- and high-magnification TEM images of Z 1, In_2_O_3_/ZnO bundles; (**d**–**f**) Low- and high-magnification TEM images of Z 2, Ag-modified In_2_O_3_/ZnO bundles.

**Figure 4:**
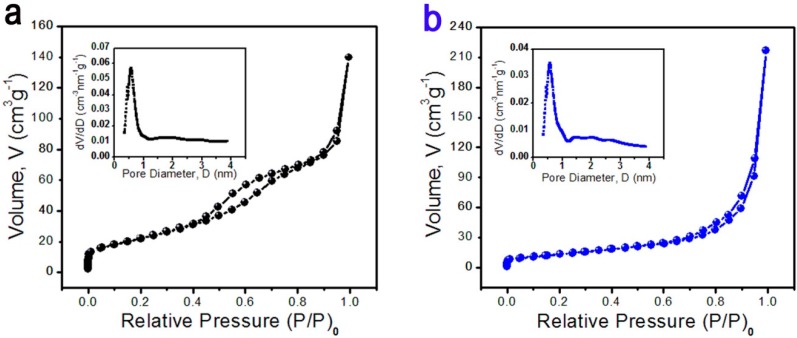
Nitrogen adsorption-desorption isotherms of the as-prepared samples, inset is the corresponding pore size distribution curve: (**a**) Z 1; (**b**) Z 2.

TEM characterization further analyzes the surface morphologies and structure of the as-prepared samples in Figure 3, by which sample Z 1 is mainly composed of ZnO and In_2_O_3_ nanoparticles with porous morphology. Unlike the Ag elements, EDX mapping results in Figure 2b, the Ag element could not be detected in high-magnification TEM images of Z 2 in Figure 3f. This may be explained by the few doping Ag element, which is too rare to be found in HRTEM. The high-magnification TEM images in Figures 3c and f illustrate that the nanoparticles are crystal and nanogaps exist between each particle, which form the porous structure for sample Z 1 and Z 2. The porous structure can also be realized by the N_2_ sorption BET and BJH algorithm showed in Figure 4 below, which is also matched to the TEM and SEM images.

The specific surface areas and the porous nature of the Z 1 and Z 2 are determined by measuring nitrogen adsorption-desorption isotherms at 77 K (Figure 4). It can be seen that both the isotherm of Z 1 and Z 2 show a hysteresis loop at relative pressure range of 0.8–1.0 P/P_0_ and the corresponding BET specific surface area is 81.54 m^2^/g for Z 1 and 50.01 m^2^/g for Z 2. In addition, the pore size distribution based on Barret-Joyner-Halenda (BJH) method is confirmed by the corresponding pore size distributions (the inset of Figures 4a and b), which reveals that the existence of mesopores in the as-prepared nanobundles.

**Figure 5:**
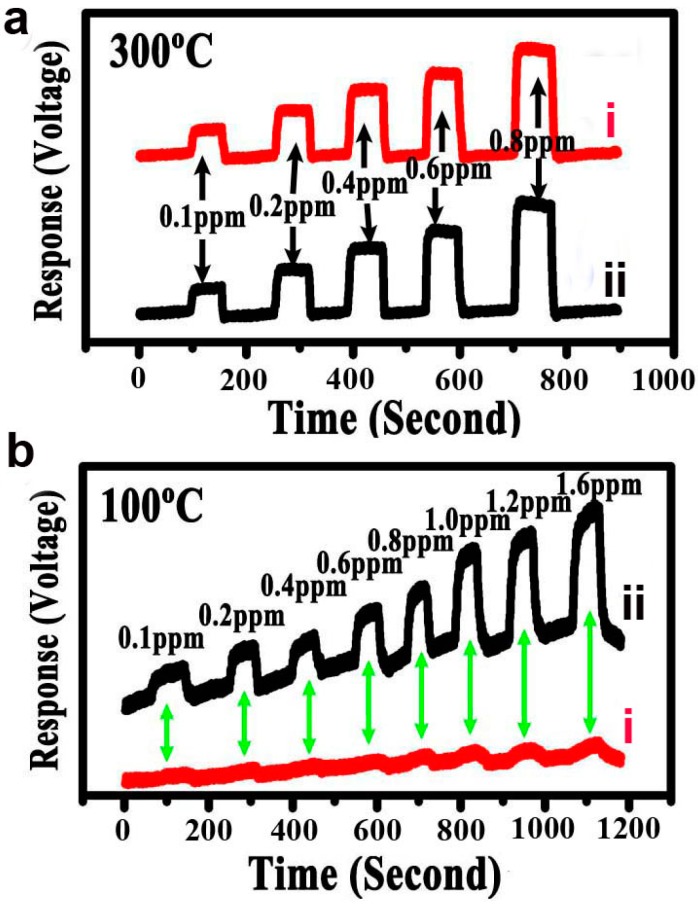
(**a**) Response curve of a sensor coated with Sample 1 (line i) and Sample 2 (line ii) at 300 °C to HCHO with the concentration of 100 ppb–800 ppb, when the HCHO gas was repeatedly introduced into the measurement system and then shut down; (**b**) Response curve of a sensor coated with Sample 1 (line i) and Sample 2 (line ii) at 100 °C to HCHO with the concentration of 100 ppb–1600 ppb.

Figure 5 shows the HCHO-sensing curve of sample Z 1 and Z 2, which was acquired by using an HCHO sensor coated with detected samples at 300 °C and 100 °C. This measurement method is the same as the reported standard [25]. The curve describes changes in the electrical signal from samples, which were caused by the reactions between the surfaces of sensor materials and the HCHO molecules [2,3,4,5,6,16,17,18]. The changes indicate that the HCHO detection limit of Sample Z 1 and Sample Z 2 are all 100 ppb at 300 °C, and the response of the HCHO sensing are all near 6 s and the recover times are near 3 s. However, when the detect temperature was set to 100 °C, the HCHO detection response of sample Z 1, which had not been modified with Ag, can’t show sharp contrast curve at low HCHO concentration. However, the HCHO detection response of Sample 2, which had been modified with Ag, showed good HCHO response with detect limitation low to 100 ppb (health standard limitation) with response time near 12 s and recover time near 6 s. It may be suspected that the good HCHO response of Sample 2 at low temperature should be related to the high electro-conductivity of silver element, which will be discussed below. The HCHO gas-sensing performance of the Ag-modified In_2_O_3_/ZnO bundles is better than the reported various nanomaterials based conductometric HCHO gas sensors, such as ZnO microoctahedrons (detection limit of 200 ppm, response time of 46 s at 400 °C) [26], ZnO nanorods (detection limit of 10 ppm, response time of >15 min with UV assistance) [27], Pt nanoparticles decorated ZnO nanowires (detection limit of 1 ppm at 265 °C) [28], SnO_2_/In_2_O_3_ nanofibers (detection limit of 10 ppm, response time of 3–5 min at 375 °C) [29], DC sputtered SnO_2_ nanowires (detection limit of 5 ppm, response time of <2 min at 270 °C) [30], thermally evaporated SnO*_x_*-Sn compound films on graphene substrate (detection limit of 10 ppm) [31], Iizuka *et al*. synthesized SnO_2_ porous film via plasma spray physical vapor deposition method. The geometry and the applied voltage of the sensors based on the SnO_2_ porous film were optimized. A Concentration of 40 ppb has been detected for films characterized by grain sizes of 37 nm containing micro and macro pores of 2 and 73 nm respectively [32]. However, complex synthesis steps and expensive equipment restrict their practical applications.

The above HCHO-sensing tests at different temperatures show that the Ag-modified In_2_O_3_/ZnO nanobundles possess good HCHO-sensing property in terms of low detection limit, short response and recover time, and especially the low-temperature-response comparing with the sample which didn’t modified with Ag. There are several possible reasons which may be responsible for the superior gas sensing performance of the porous Ag-modified In_2_O_3_/ZnO nanocomposites: (1) The Ag-modified In_2_O_3_/ZnO with porous feature can provide more active sites to react with target gas molecules, which may lower the detect limitation [25]; (2) The nanogaps between each nanoparticle can increase the gas diffusion speed, which are responsible for response and recovery time; (3) The addition of Ag element with good electrical conductivity can increase the sensors’ electronic conductivity, therefore leading to higher response ability [21,22]; (4) The porous Ag-modified In_2_O_3_/ZnO nanobundles with the In_2_O_3_/ZnO micro/nano bundle structures can prevent the aggregation of nanocrystals when compared to the monodispersed case, which is favorable for preventing the undesirable aggregation of these nanobuildings and ensure the stability of the sensors. Therefore, abundant mesopores with large specific surface area and modified with Ag element are of importance for the HCHO-sensing properties, which is the main reason that Ag-modified In_2_O_3_/ZnO nanobundles sample shows superior HCHO sensor performance.

## 4. Conclusions

In summary, Ag-modified In_2_O_3_/ZnO nanobundles with porous structure have been designed and synthesized by a hydrothermal method continuing with dehydration process. When used as the sensor materials for HCHO-sensing, the as-prepared samples showed good HCHO sensing properties with low HCHO detection limit, short response and recovery time. The porous structure, high specific surface area, and the content of Ag element, have been investigated, respectively. The HCHO-detection limit of 100 ppb (parts per billion) and the response and recover times as short as 6 s and 3 s, respectively, at 300 °C, and the detection limit of 100 ppb, response time of 12 s and recover times of 6 s at 100 °C, matching the health standard. The good sensor performance may be attributed to the porous feature, the addition of Ag element, and the special designed In_2_O_3_/ZnO nanobundles structure.

## Supplementary Files

Supplementary File 1
