# Supplementary material for: Ag-Modified In2O3/ZnO Nanobundles with High Formaldehyde Gas-Sensing Performance
Source: Sensors (Basel). 2015 Aug 14;15(8):20086–96. doi: 10.3390/s150820086 (PMC4570411; doi:10.3390/s150820086)
Supplement: Supplementary File 1 [file sensors-15-20086-s001.pdf]

OPEN ACCESS
sensors
ISSN 1424-8220
www.mdpi.com/journal/sensors

Supplementary Information

Ag-Modified In_2O_3/ZnO Nanobundles with High Formaldehyde Gas-Sensing Performance. *Sensors* 2015, *15*, 20086-20096

Fang Fang [1], Lu Bai [2], Dongsheng Song [1], Hongping Yang [1], Xiaoming Sun [3], Hongyu Sun [1,*] and Jing Zhu [1,*]

1 Beijing National Center for Electron Microscopy, School of Materials Science and Engineering, The State Key Laboratory of New Ceramics and Fine Processing, Key Laboratory of Advanced Materials (MOE), Tsinghua University, Beijing 100084, China; E-Mails: ff99@mail.tsinghua.edu.cn (F.F.); todongsheng@126.com (D.S.); hpyang1989@163.com (H.Y.)

2 National Center for Nanoscience & Technology University of Chinese Academy of Sciences, No. 11 First North Road, Zhongguancun, Beijing 100190, China; E-Mail: whitelu@mail.tsinghua.edu.cn

3 State Key Laboratory of Chemical Resource Engineering, Beijing University of Chemical Technology, Beijing 100029, China; E-Mail: sunxm@mail.buct.edu.cn

* Authors to whom correspondence should be addressed; E-Mails: hysuny@mail.tsinghua.edu.cn (H.S.); jzhu@mail.tsinghua.edu.cn (J.Z.); Tel.: +86-10-6277-3998 (H.S.).

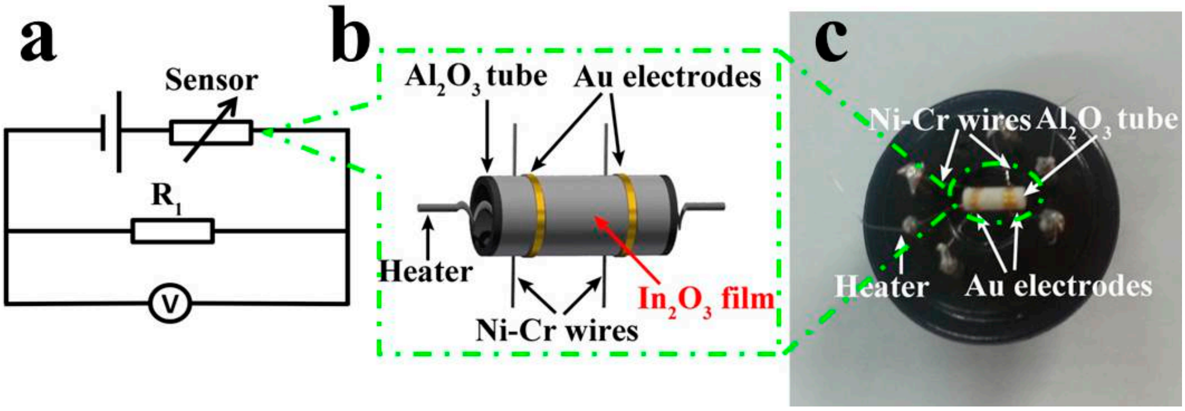

Figure S1. (**a**) Equivalent circuit of the sensor with the measuring system, in which R_1 is a small resistor in the system [1]; (**b**) Schematic image (not to scale) of the structure of an HCHO sensor, in which the small Al_2O_3 tube is coated with as-prepared sample and the resistive heater inside the Al_2O_3 tube is used to control the sensors' temperature; (**c**) Optical image of an HCHO sensor that has been mounted on the sensor holder of the measuring system.

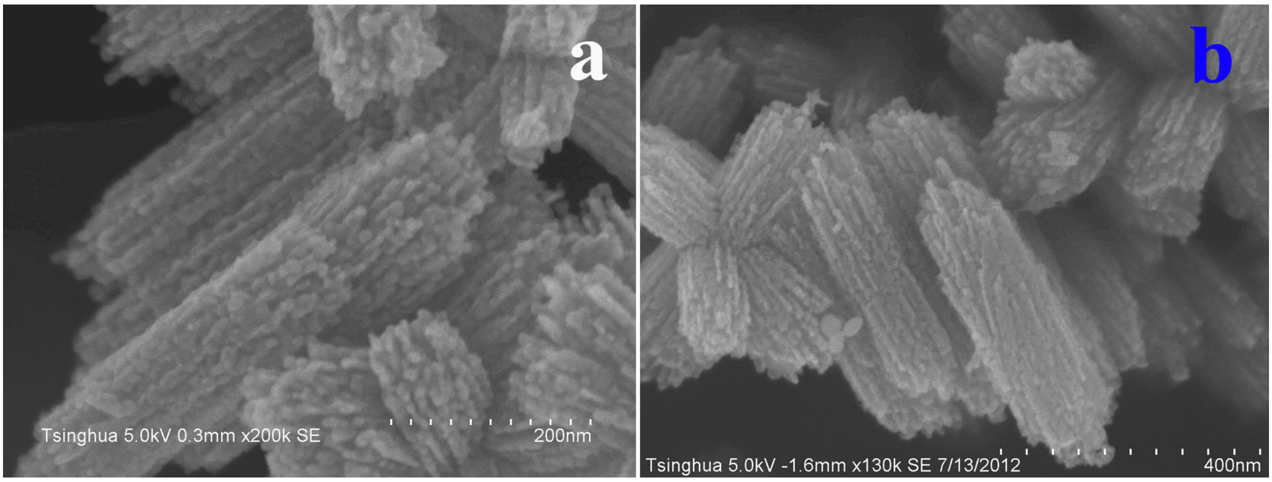

Figure S2. SEM images of samples after aging at 300 °C for 2 h, (**a**) sample Z 1 (**b**) sample Z 2.

References

1. Fang, F.; Bai, L.; Sun, H.Y.; Kuang, Y.; Sun, X.M.; Shi, T.; Song, D.S.; Guo, P.; Yang, H.P.; Zhang, Z.F.; *et al*. Hierarchically porous indium oxide nanolamellas with ten-parts-per-billion-level formaldehyde-sensing performance. *Sens. Actuators B* **2015**, *206*, 714–720.

© 2015 by the authors; licensee MDPI, Basel, Switzerland. This article is an open access article distributed under the terms and conditions of the Creative Commons Attribution license (http://creativecommons.org/licenses/by/4.0/).
